# *Laminaria japonica* Extract Enhances Intestinal Barrier Function by Altering Inflammatory Response and Tight Junction-Related Protein in Lipopolysaccharide-Stimulated Caco-2 Cells

**DOI:** 10.3390/nu11051001

**Published:** 2019-05-01

**Authors:** Hyo-Seon Yang, Fawaz G Haj, Myoungsook Lee, Inhae Kang, Guiguo Zhang, Yunkyoung Lee

**Affiliations:** 1 Department of Food Science and Nutrition, Jeju National University, Jeju 63243, Korea; gytjs414@naver.com (H.-S.Y.); inhaek@jejunu.ac.kr (I.K.); 2 Department of Nutrition, University of California Davis, One Shields Ave, Davis, CA 95616, USA; fghaj@ucdavis.edu; 3 Department of Food and Nutrition, Sungshin Women’s University, Seoul 01133, Korea; mlee@sungshin.ac.kr; 4 Department of Animal Nutrition, Shandong Agricultural University, Tai’an 271018, China; zhanggg@sdau.edu.cn

**Keywords:** Caco-2 cells, *Laminaria japonica*, fermentation, anti-inflammatory, gut permeability, inflammatory bowel disease

## Abstract

In the normal physiological state, intestinal epithelial cells act as a defensive frontline of host mucosal immunity to tolerate constant exposure to external stimuli. In this study, we investigated the potential anti-inflammatory and gut permeability protective effects of *Laminaria japonica* (LJ) water extract (LJE) and three types of fermented *Laminaria japonica* water extracts (LJE-F1, LJE-F2, and LJE-F3) in lipopolysaccharide (LPS)-stimulated Caco-2, human intestinal epithelial cells. All four extracts significantly decreased the production of nitric oxide and interleukin-6 induced by LPS stimulus. In addition, LJE and the three types of LJE-Fs also inhibited LPS-induced loss of monolayer permeability, as assessed by changes in transepithelial electrical resistance. All four LJ extracts significantly prevented the inhibition of the protein levels of occludin, whereas LJE, LJE-F1, and LJE-F3 significantly attenuated the reduction in phosphorylation of adenosine monophosphate-activated protein kinase compared with the LPS-treated group in Caco-2 cells. In conclusion, LJE and its fermented water extracts appear to have potential gut health-promoting effects by reducing inflammation and partially regulating the tight junction-related proteins in human intestinal epithelial cells. Thus, additional studies are warranted to evaluate *Laminaria japonica* as a therapeutic agent for inflammatory bowel diseases.

## 1. Introduction

The gastrointestinal (GI) tract is constantly challenged by numerous external stimuli, and the epithelial cells function as a frontline defense barrier from those potential xenobiotics by closely regulating the tight junction (TJ). Disrupted TJs that lead to increased gut permeability cause barrier malfunction of epithelial cells—the so-called ‘leaky gut’. In this physiologically vulnerable state, various pathogens, antigens, and endotoxins can easily penetrate the epithelial lining of the GI tract and may provoke abnormal intestinal inflammation and the development of chronic inflammatory diseases, such as inflammatory bowel disease (IBD) [1]. The increased incidence of IBDs is a major public health problem, because it affects patients’ quality of life enormously [2]. The treatments for IBDs are mainly to manage the inflammatory status with different drug categories including aminosalicylates, corticosteroids, anti-tumor necrosis factor alpha drugs, antibiotics, probiotics, and immunosuppressants, which sometimes are inadequate [3]. In recent decades, looking for alternative medicines from natural sources has received much attention to improve the lack of desirable efficacy and poor tolerability of the current drugs for treating IBDs [4,5,6].

In particular, intestinal epithelial cells play a critical role as a defensive frontline of host mucosal immunity to tolerate constant exposure to external stimuli including Gram-negative bacteria [7]. Caco-2, a human intestinal epithelial cell line, is the most well-established cell line for investigating intestinal barrier integrity and function [8]. Various stimuli have been used to induce inflammatory status to mimic a similar physiological condition to that in Caco-2 cells; one of them is a lipopolysaccharide (LPS), a principal component of the outer membrane of Gram-negative bacteria [9,10,11]. LPS is known to trigger an inflammatory signaling cascade via activating Toll-like receptor (TLR)-4, a member of the transmembrane protein family that functions as a pattern-recognition receptor [12]. Upon LPS stimuli, epithelial cells produce proinflammatory cytokines to disrupt TJs, leading to increased gut permeability [10,13].

Various seaweeds are commonly consumed as foods worldwide, especially in countries in Asia. *Laminaria japonica* (LJ) is a popular, edible, brown seaweed that is present in high quantities on Jeju Island, Korea [14]. In general, LJ contains soluble fibers, such as alginate and fucoidan, as well as fat-soluble components, such as fucoxanthin and fucosterol, along with minerals such as magnesium, iodine, calcium, iron, and zinc [15,16]. Its biological functions have been extensively investigated in both in vitro and in vivo models. LJ extracts have been shown to have anti-obesity, antidiabetic, anti-inflammatory, anti-oxidant, and anticancer effects in a number of studies [15,17,18,19]. Furthermore, the application of fermentation to LJ has attracted attention as a tool to enhance its biological functions, as well as attenuate its potential toxicity [20,21,22,23]. Fermentation with microorganism(s) has been shown to improve the extraction of bioactive substances from original materials [20,23,24]. For example, Kang et al. [20] reported that the administration of *Lactobacillus brevis*-fermented LJ (1.5 g/day for four weeks) enhanced the antioxidant defense system in humans. Fermented LJ extract protected oxidative-stressed porcine kidney epithelial cells. In addition, Lin et al. [21] demonstrated the enhanced anti-inflammatory effects of LJ fermented by *Bacillus subtilis* (200 μg/mL), whereas LJ water extract did not cause a significant decrease compared with the LPS-stimulated group in consideration of nitric oxide (NO) production induced by LPS in mouse macrophages, RAW 264.7 cells.

Thus, most previous studies on fermented *Laminaria japonica* have been limited to its antioxidant effects and anti-inflammatory effects. In the present study, we utilized Caco-2, a well-studied human intestinal epithelial cell line, for the investigation of in vivo intestinal epithelial barrier integrity and function [8], to evaluate the potential protective effect and mechanism(s) of LJ water extract (LJE) and/or three types of fermented LJEs by probiotic strains on LPS-stimulated TJ destruction in human intestinal epithelial cells, which may provide a novel approach for the treatment of IBDs.

## 2. Materials and Methods

### 2.1. Preparation of Extracts from Laminaria japonica

**Figure 1:**
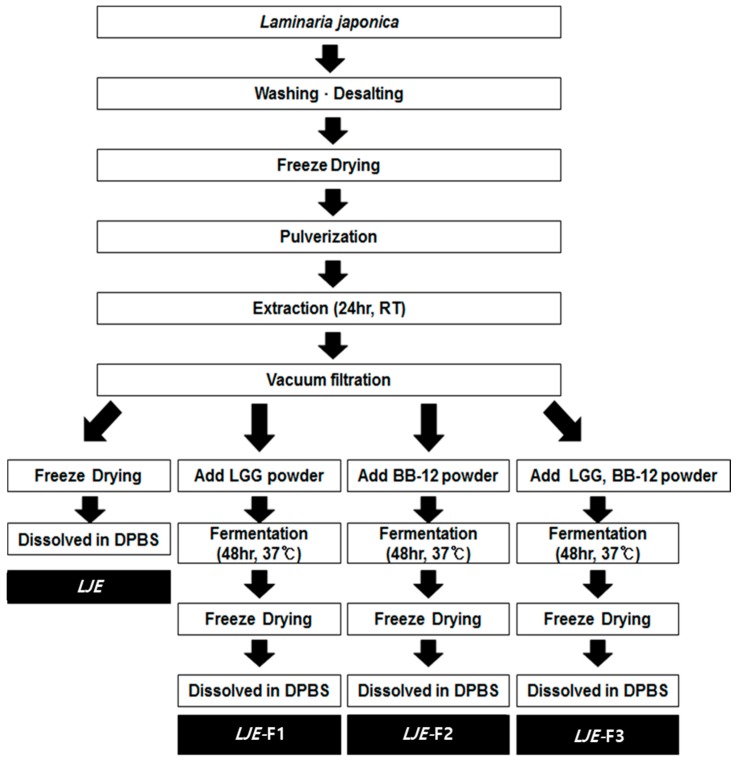
A flow diagram of *Laminaria japonica* (LJ) water extract (LJE) and fermented LJ water extract (LJE-F1, -2, and -3) preparation. Abbreviations: LGG; *Lactobacillus rhamnosus* LGG, BB-12; *Bifidobacterium animalis* ssp. *lactis* BB-12; DPBS, Dulbecco’s phosphate-buffered saline; and RT, room temperature.

Raw *Laminaria japonica* was purchased from a traditional market in Jeju and processed into powder, as previously described [17]. The detailed sample preparation from LJE and fermented LJEs is described in Figure 1. Briefly, the freeze-dried LJ powder (3 g) was extracted using 200 mL double-distilled water in a shaking water bath for 24 h at room temperature, followed by vacuum filtration. For LJE, the solution was freeze dried and dissolved in Dulbecco’s phosphate-buffered saline (DPBS, Gibco, Gaithersburg, MD, USA). For the LJE-Fs, the vacuum-filtered solution (100 mL) was mixed with 6 × 10^10^ of *Lactobacillus rhamnosus* LGG (LGG, 3 × 10^10^/g, CHR-Hansen, Roskilde, Denmark) for LJE-F1, *Bifidobacterium animalis* ssp. *lactis* BB-12 (BB-12, 3 × 10^10^/g, CHR-Hansen) for LJE-F2, or LGG and BB-12 for LJE-F3. Both LGG and BB-12 are well-established probiotic strains [25], provided by the research and development center of Maeil Dairies Co. (Pyeongtaek, Gyeonggi-do, Korea). Based on the preliminary experiment results and a published literature search [21,24,26,27,28,29], to optimize the fermentation process, fermentation of LJ was carried out at 37 °C for 48 h and then terminated at ≤ pH 4.0, followed by heat treatment at 120 °C for 20 min and freeze drying. The final powder of three LJE-Fs was dissolved in DPBS and filtered for cell culture experiments.

### 2.2. Measurement of pH, Sugar, and Reducing Sugar Contents

Alterations of LJEs by the fermentation process were measured in pH, sugar, and reducing sugar contents of extracts. The phenol‒sulfuric acid method was utilized to check changes in total sugar contents before and after fermentation of LJE [30]. Briefly, 10-μL samples were mixed with 10 μL of 5% phenol solution, and then, 50 μL 18 M H_2_SO_4_ was added. The reaction mixture was set at room temperature for 30 min, and the absorbance was measured at 490 nm. Based on a D-Glucose standard solution (Katayama Chemical, Osaka, Japan), the total sugar contents were calculated. Reducing sugar contents in samples were measured by the dinitrosalicylic acid (DNS) method [31]. Then, 1 mL of diluted sample was mixed well with 2 mL of DNS (3,5-dinitrosalicylic acid, 2 M NaOH, and potassium sodium tartrate) and incubated in boiling water for 15 min. Absorbance was measured at 540 nm, and D-glucose was also used as a standard.

### 2.3. Total Polyphenol Contents

The Folin‒Denis method was used to determine the total polyphenol contents of LJE samples before and after fermentation [32]. First, 50-mL samples were mixed with 50 μL of 1 M Folin‒Ciocalteu’s phenol reagent (Merck KGaA, Burlington, MA, USA) and incubated at room temperature for 6 min. The reaction was further processed by adding 100 μL of 2% Na_2_CO_3_ and incubating for 30 min. Absorbance was measured at 720 nm, and gallic acid (Sigma-Aldrich, St. Louis, MO, USA) was used as a standard compound.

### 2.4. Cell Culture

Caco-2 cells were purchased from the Korean Cell Line Bank (Seoul, Korea) and cultured in Dulbecco’s modified Eagle’s (DMEM-low glucose) supplemented with 10% (*v/v*) fetal bovine serum (FBS), 1% (*v/v*) penicillin/streptomycin, and 1% (*v/v*) of 100× non-essential amino acid. Caco-2 cells were refed with fresh medium every two days during cell growth and differentiation under a humidified atmosphere of 5% CO_2_ at 37 °C. For the experiments, cells were used 16‒21 days after reaching confluence to allow for differentiation into intestinal epithelial cells. Cells were used between passages 10 and 20. All cell culture media and reagents were from Gibco (Gaithersburg, MD, USA).

### 2.5. Cell Viability Assay

Cell viability was determined by 3-(4,5-dimethyl thiazol-2-yl)-2,5-diphenyl tetrazolium bromide (MTT) assay [33]. Briefly, Caco-2 cells were plated in a 24-well plate at a concentration of 1 × 10^5^ cells/well and stabilized for 24 h. Then, cells were treated with different concentrations of LJE or LJE-Fs (0, 50, 100, 200, or 400 μg/mL) for 24 h. At the end of treatment, the culture media was replaced, and 100 μL of 2 mg/mL MTT reagent was added for another 4 h at 37 °C. Then, 100 μL of DMSO (dimethyl sulfoxide) was added to each well to dissolve the formazan product from cells and the absorbance was measured at 540 nm using a microplate reader (Molecular Devices, San Jose, CA, USA).

### 2.6. Nitric Oxide Production Measurement

Alteration of nitric oxide (NO) production by LJE and/or LJE-Fs treatment was measured in collected cultured Caco-2 media using the Griess Reagent system [34]. Caco-2 cells were seeded at 0.25 × 10^6^ cells/well (24-well plate, Corning, Oneonta, NY, USA). The cell culture medium was changed every 2 days until the cells were fully differentiated (14–21 days). Next 100 µg/mL of LJE and LJE-Fs were applied to cells for 6 h. Then, 1 µg/mL of lipopolysaccharide (Sigma-Aldrich) was added, and the solution incubated for another 24 h [9]. At the end of treatment, 100 μL of cultured medium was transferred to a 96-well plate, and the same volume of Griess reagent (Sigma-Aldrich) was added. The reaction was carried out for 10 min at room temperature, and absorbance was measured by a microplate reader at 540 nm. Relative nitric oxide (NO) production was calculated based on the LPS-treated group, which was considered to be 100%.

### 2.7. Transepithelial Electrical Resistance

To determine the effects of LJE and LJE-Fs on the TJ stabilization of Caco-2 cells, transepithelial electrical resistance (TEER) assay was performed according to a previous study with modifications [35]. Cells were differentiated into polarized monolayers by culture on 12-transwell plates (Corning, Oneonta, NY, USA) at a seeding density of 0.25 × 10^6^ cells/well. The volume of medium added to the upper and lower compartment was 0.5 mL and 1.5 mL, respectively. TEER was measured using a Millicell-ERS resistance system (Millipore, MA, USA) that includes a dual-electrode volt-ohm-meter. TEER was calculated as TEER Ωcm^2^ = (target TEER value (T_24_) − control TEER value)/(target TEER value (T_0_) − control TEER value) (Ω) × A (cm^2^); being, target TEER value, transmembrane resistance; control TEER value, intrinsic resistance of a cell-free media; and A, the surface area of the membrane in cm^2^. Cells were rinsed with DPBS, and then, Hanks’ Balanced Salt Solution (HBSS, Gibco, Gaithersburg, MD, USA) was added in both compartments. Cells were treated with LJE or LJE-Fs (100 μg/mL), which was added to the upper compartment for 6 h, and LPS (1 μg/mL) was subsequently added to the lower compartment for an additional 24 h. The TEER values were measured at 0 h (T_0_) and the end of treatment (T_end_).

### 2.8. Western Blot Analysis and ELISA

Cells in six-well plates at a seeding density of 1 × 10^6^ cells/well were cultured for 14‒21 days. Cells were then pre-incubated for 6 h with 100 μg/mL LJ or LJE-Fs and incubated for 24 h in the absence or presence of LPS (100 μg/mL). Collected media was used to measure inflammatory cytokines by ELISA kit (TNF-α and IL-6; BD PharMingen, San Diego, CA, USA) according to the manufacturer’s protocol. After rinsing with DPBS three times, cells were lysed by adding 100 μL of lysis buffer (20 mM Tris-HCL (pH 7.4), 5 mM Na_4_P_2_O_7_, 10 mM NaF, 100 mM NonidetP-40, 1% NaVO_4_, and 0.5% EZ block), and centrifugation was carried out for 15 min at 14,000 rpm and 4 °C. Proteins were quantified using a protein assay kit (Thermo Fisher Scientific, Lombard, IL, USA). Proteins (50 μg/24 μL) were then separated by 10% SDS-PAGE (SDS polyacrylamide gel electrophoresis) at 80 V for 1.5 h and then 120 V for 0.5 h and transferred to nitrocellulose membranes (Amersham Co., Freiburg, Germany) at 300 mA for 1.5 h. Membranes were then blocked with 5% blocking buffer (Bio-rad, Hercules, CA, USA) for 2 h followed by the indicated antibody (phospho-AMPK (adenosine monophosphate (AMP)-activated protein kinase)α (Thr 172), AMPKα, GAPDH (1:1000, Cell signaling technology, MA, USA), occludin (65 kDa, 1:200, Santa Cruz, CA, USA)) at 4 °C overnight and horseradish peroxidase-coupled anti-species antibodies at room temperature for 1 h. Proteins were visualized by enhanced chemiluminescence and quantified by densitometry using Fusion solo (Vilber Lourmat, Baden-Württemberg, Germany). The intensity of immunoblot analysis was quantified by ImageJ software (National Institutes of Health, Bethesda, MD, USA) [36].

### 2.9. Statistical Analysis

Statistical analysis was performed using ANOVA (one-way analysis of variance) followed by Tukey’s multiple comparison test. All the data are expressed as the mean ± SEM (standard error of the mean). Differences of *p* < 0.05 were considered significant (GraphPad Prism version 6.0, La Jolla, CA, USA).

## 3. Results

### 3.1. Changes of Chemical Characteristics of Laminaria japonica by Fermentation

Changes by fermentation, including pH, sugar, and reducing sugar contents and total polyphenol contents of LJEs, are shown in Table 1. Before the fermentation began, the pH of LJ extracts LJE-F1, LJE-F2, and LJE-F3 was 7.30, 7.31, and 7.34, respectively. After 48 h of fermentation, the pH of LJEs went down to 4.06, 4.01, and 3.95, respectively. Total sugar and reducing sugar contents were significantly decreased by the 48-h fermentation in all three types of LJE-Fs. In addition, the total polyphenol contents in the LJE-Fs were not decreased compared with LJE.

### 3.2. Effect of LJE and LJE-Fs on Cell Viability in Caco-2 Cells

**Figure 2:**
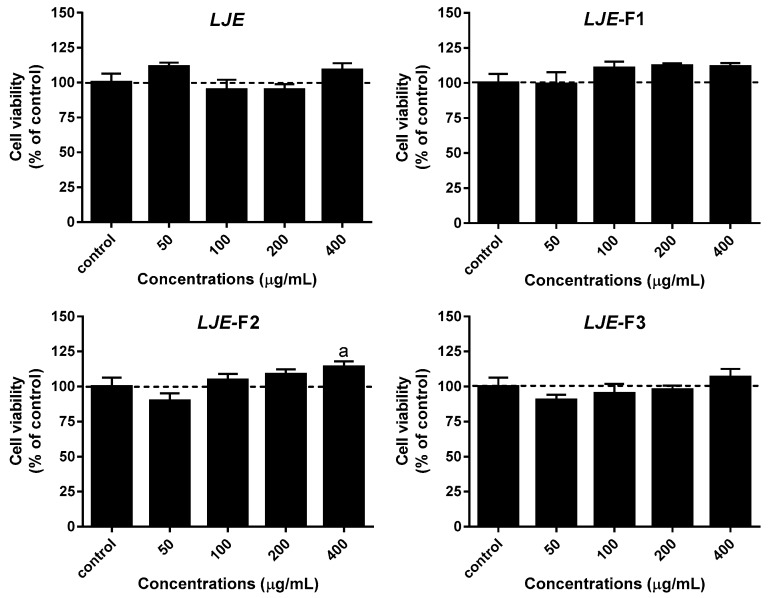
Effects of LJE and LJE-Fs on Caco-2 cell viability. Caco-2 cells (1 × 10^5^ cells/well) were seeded in a 24-well plate and treated with LJE or LJE-Fs (0, 50, 100, 200, or 400 μg/mL) for 24 h. Values are mean ± SEM of four independent experiments (*n* = 3–6). ^a^ *p* < 0.05 compared with the control by one-way ANOVA with Tukey’s comparisons test.

To determine optimal concentrations of LJE and LJE-Fs to treat Caco-2 cells, MTT assay was performed with various concentrations of LJE and LJE-Fs. The cellular toxicity of all four types of LJE was evaluated in Caco-2 cells by MTT assay, as shown in Figure 2. Caco-2 cells were treated with 0, 50, 100, 200, and 400 μg/mL of LJE or LJE-Fs for 24 h. There was no significant reduction in cell viability by different doses of LJE or LJE-Fs. In the case of LJE-F2, cell viability indeed significantly increased at 400 μg/mL compared with the control group (*p* < 0.05). Based on this cellular toxicity assay as well as our previous studies in various cell culture models with seaweed extracts [17,37], 100 μg/mL of LJE or LJE-Fs where there was a minimum alteration (less than 7%) on cell viability compared with the control was used for the rest of the experiments.

### 3.3. LJE and LJE-Fs Decreased the Production of Nitric Oxide by LPS Stimulus in Caco-2 Cells

**Figure 3:**
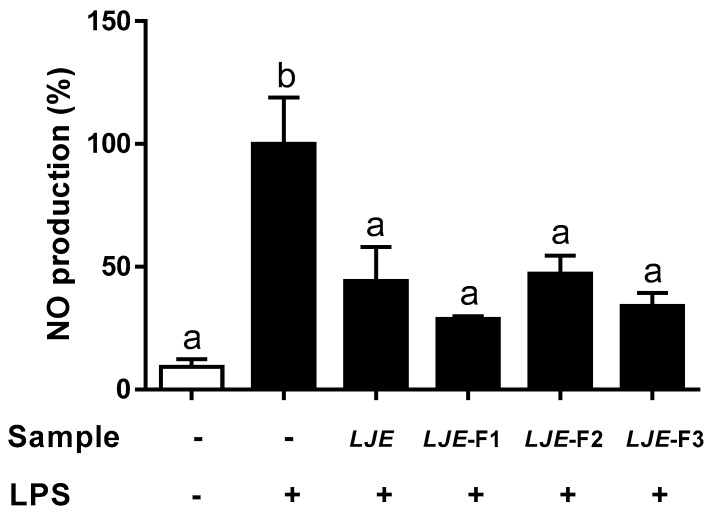
Effects of LJE and LJE-Fs on nitric oxide (NO) production in the lipopolysaccharide (LPS)-stimulated Caco-2 cells. Caco-2 cell monolayers were incubated for 6 h with 100 μg/mL LJE, LJE-F1, LJE-F2, or LJE-F3. Then, 1 μg/mL LPS was added, and the cells were incubated for an additional 24 h. Values are mean ± SEM of three independent experiments. Values that do not share the same superscript are significantly different by one-way ANOVA with Tukey’s comparisons test (*p* < 0.05).

To understand how LJE and LJE-Fs act as an anti-inflammatory substance, we investigated the inhibition of NO production by LJ extracts in LPS-stimulated Caco-2 cell monolayers. The capacity of LJE and LJE-Fs to attenuate NO production induced by LPS in Caco-2 cells was shown in Figure 3. Compared with the NO production from the LPS-stimulated group as a control (100%), all four LJEs significantly decreased NO productions, 44.24% by LJE, 28.73% by LJE-F1, 47.24% by LJE-F2, and 34.04% by LJE-F3. Thus, LJE and the three types of LJE-Fs appeared to significantly decrease NO production by LPS stimulus in Caco-2 cells.

### 3.4. LJE and LJE-Fs Protected Caco-2 Cell Monolayers from Endotoxin Stimulus

**Figure 4:**
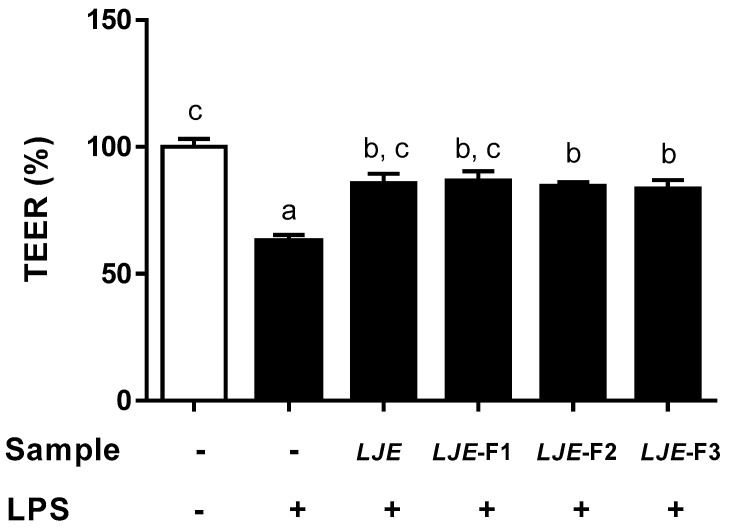
Effects of LJ and LJE-Fs on permeabilization of Caco-2 cell monolayers measured by transepithelial electrical resistance (TEER (%). Caco-2 cell monolayers were preincubated with 100 μg/mL LJE, LJE-F1, LJE-F2, or LJE-F3 for 6 h, and the cells were incubated for an additional 24 h with 1 μg/mL LPS. Caco-2 cell monolayer permeability was evaluated by measuring TEER. The results are shown as mean ± SEM of three independent experiments. Values that do not share the same superscript are significantly different by one-way ANOVA with Tukey’s comparisons test (*p* < 0.05).

Next, we evaluated the preventive effects of LJE and LJE-Fs on intestinal epithelial barrier function disrupted by LPS stimulus in polarized Caco-2 cell monolayers by measuring TEER (Figure 4). Time zero (T_0_)-TEER values were measured before 100 μg/mL LJE or LJE-Fs were added to the upper compartment of the Caco-2 cell monolayer. Followed by LPS stimulus for 24 h, the TEER values were evaluated at the end of treatment. Values were expressed as percentages compared with the control group. As a result, the LPS-simulated group showed a significant increase in the permeabilization of Caco-2 monolayers as TEER values were significantly decreased to 62.90% of that of the control (100%). On the other hand, LJE- and LJE-Fs-treated groups all protected Caco-2 cell monolayers, as indicated by the significantly increased TEER values (LJE: 85.70%; LJE-F1: 86.76%; LJE-F2: 84.64%; and LJE-F3: 83.58%) compared with the LPS-treated group.

### 3.5. LJE and LJE-Fs Significantly Attenuate IL-6 Production Induced by LPS Stimulus in Caco-2 Cells

To further evaluate the anti-inflammatory effects of LJE and LJE-Fs, the production of TNF-α and IL-6 was measured in Caco-2 cell monolayers. LPS stimulation increased both TNF-α (21.90 pg/mL) and IL-6 (5.47 pg/mL) in Caco-2 cells compared with the no LPS-treated group, which showed no detectable ranges of TNF-α and IL-6 (Table 2). Only LJE and LJE-F1 significantly decreased the LPS-induced TNF-α production, whereas LJE-F2 and LJE-F3 did not significantly decrease TNF-α production in Caco-2 cells. On the other hand, all four extracts, LJE and LJE-Fs, appeared to significantly attenuate LPS-induced IL-6 production (5.47 pg/mL)—4.16 pg/mL by LJE, 0.92 pg/mL by LJE-F1, 0.91 pg/mL by LJE-F2, and 0.77 pg/mL by LJE-F3—in Caco-2 cells compared with the LPS-treated group. LJE-Fs appeared to have superior effects on decreasing IL-6 production compared with LJE itself.

### 3.6. LJE and LJE-Fs Differentially Promoted Tight Junction (TJ)-Related Protein Expression in Caco-2 Cells

**Figure 5:**
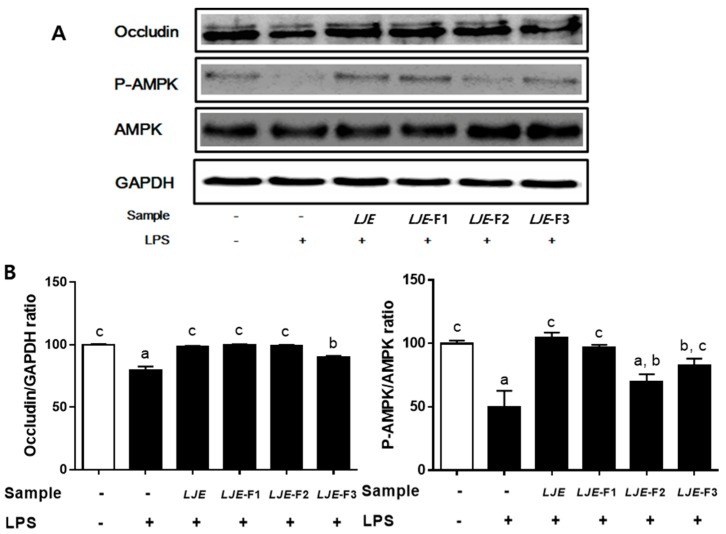
LJE and LJE-Fs differentially promoted intestinal epithelial barrier function via upregulation of the tight junction (TJ) in Caco-2 cell monolayers. (**A**) Representative immunoblot analysis of occludin, phospho-adenosine monophosphate-activated protein kinase (P-AMPK), total AMPK, and glyceraldehyde-3-phosphate dehydrogenase (GAPDH); (**B**) intensity of occludin was normalized to GAPDH, and intensity of P-AMPK was normalized to total AMPK to account for apparent differences and confirm statistical significance. Caco-2 cells were pre-incubated for 6 h with 100 μg/mL LJE or LJE-Fs and then incubated for 24 h in the absence or presence of LPS (100 μg/mL). Values are mean ± SEM of three independent experiments. Values that do not share the same superscript are significantly different by one-way ANOVA with Tukey’s comparisons test (*p* < 0.05).

Lastly, two TJ-related proteins, occludin and AMPK, were evaluated to investigate a potential mechanism of preventive effects of LJE and LJE-Fs on intestinal barrier function. As a result, pretreatment with all LJE and LJE-Fs attenuated the reduction in protein expression of occludin, a TJ protein that plays a critical role in regulating permeability of epithelial cells, while LPS reduced the protein expression of occludin compared with the non-LPS-treated group in Caco-2 cell monolayers (Figure 5). In addition, LJE, LJE-F1, and LJE-F3 significantly prevented a decrease of phosphorylation of AMPK, a therapeutic target in intestinal diseases [38] by LPS stimulus in Caco-2 cell monolayers. These results suggest that LJE and LJE-Fs differentially promoted TJ-related proteins, such as occludin and AMPK that may lead to enhancing the intestinal epithelial barrier function.

## 4. Discussion and Conclusions

The major forms of IBDs include Crohn’s disease (CD), ulcerative colitis (UC), and unclassified IBD. Although causes of IBDs are not fully understood yet, the association of IBDs with an epithelial barrier dysfunction characterized by impaired nutrients absorption, as well as mucosal barrier functions, has been appreciated [1,2]. A defective intestinal epithelial TJ barrier is an important pathogenic factor to determine gut permeability, because penetration of luminal noxious molecules via the disrupted intestinal TJ induces a perturbation of the mucosal immune system and inflammation, which subsequently act as a trigger for the development of intestinal diseases. The roles of extracellular factors affecting the integrity of intestinal TJ, such as cytokines, pathogens, and even food factors, have been extensively studied [3,4,5].

*Laminaria japonica*, a type of brown seaweed, has been studied intensively due to its various health benefits, such as anti-oxidant, antidiabetic, anti-inflammatory, and anti-obesity properties, which potentially prevent and/or treat lifestyle-related diseases [14,15,17,18,39]. In a few countries in Asia, LJ is not only a popular edible seaweed but has also been an alternative medicine to treat gastrointestinal issues since ancient times [40]. Based on our previous studies, among the various brown seaweeds that we have screened, LJ appears to have the most potent health benefits, including antidiabetic and anti-inflammatory effects in in vitro and in vivo models [17,18]. Recently, fermentation has been applied to improve the bioactivity of the seaweed extraction procedure [20,21,22,23]. However, the reported effects of fermented LJ extraction are limited to its anti-inflammatory and anti-oxidant effects so far [20,21,27,28]. In this study, we investigated the capacity of LJ water extract and three types of fermented LJ extracts (LJE-F1, LJE-F2, and LJE-F3) by two natural residents of the gastrointestinal tract and probiotic strains, LGG and BB-12, to inhibit the permeabilization of Caco-2 cell monolayers and their potential mechanism(s).

It has been reported that fermentation not only improves the nutrient contents of food via the biosynthesis of vitamins, amino acids, and proteins but also promotes the digestibility and bioavailability of foods [23,24,29,41]. In this study, LJ fermentation by probiotic strains, LGG or BB-12, or a combination of both resulted in some alterations in total sugar and reduction of sugar levels in LJE-Fs. Fermentation is a biochemical reaction that metabolizes high-molecular-weight organic compounds to smaller and simpler molecules. Therefore, it is not surprising that the total sugar and reducing sugar levels in LJE-Fs were decreased significantly, which may be a sign of fermentation. A previous report by Eom et al. (2010) also showed a similar result in that LJ extract fermented *S. cerevisiae* also caused a significant reduction in total sugar levels compared with LJ extract [27].

In addition, microorganisms during fermentation utilize dietary carbohydrates, especially soluble dietary fiber, as substrates and produce short-chain fatty acids (SCFAs), primarily acetate, propionate, and butyrate, as end products [42]. In fact, of the brown seaweeds, LJ contains as much as 80% soluble dietary fiber, such as alginate, fucans, fucoidans, and laminarins, among its total dietary fiber [43]. Although we did not measure SCFA contents in our LJE and LJE-Fs, it is worth noting that SCFA in LJE-Fs should be taken into account for its superior effect observed in the present study, such as the anti-inflammatory effect achieved by further reducing LPS-induced IL-6 production of LJE-Fs in Caco-2 cells. As shown in Figure 2, all three LJE-Fs increased the cell viability of Caco-2 cells, which we were not able to observe in the LJE-treated cells. Furthermore, the reduction of LPS-induced IL-6 production by LJE-Fs was significantly higher than that by LJE in Caco-2 cells. It has been reported that SCFAs indeed play a critical role in the growth of intestinal mucosal cells and Caco-2 cells [42,44], as well as in the modulation of the inflammatory response in human neutrophils and epithelial cells [45,46].

LJ is also a rich source of polyphenols, which have been considered to be an alternative medicine to treat IBDs [47,48]. Although the precise mechanism is unclear, polyphenols, such as quercetin, kaempferol, myricetin, genistein, catechin, and curcumin enhance barrier integrity in intestinal Caco-2 cells [49]. The total polyphenol contents were not significantly altered by fermentation (LJE vs. LJE-Js) in this study. Unlike our findings, most studies regarding the changes of polyphenol levels in seaweed by fermentation demonstrated increased polyphenol levels after fermentation. For example, the fermentation of *Eisenia bicyclis*, a type of brown seaweed, by yeast strain *Candida utilis*, enhanced the levels of phenolic compounds [28], and LJ extract fermented by *Aspergillus oryzae* appeared to have more phenolic compounds compared to the LJ extract itself [29]. In addition, the fermentation of brown seaweed *Sargassum siliquanstrum* by various lactic acid bacteria, including *Lactobacillus* sp., increased polyphenol levels as well [26]. While it is difficult to compare these results directly, various factors including type(s) of microorganisms, concentration, and components of substrate depending on its solvent, duration, and temperature of fermentation, as well as the time of year the seaweed was harvested, might account for the discrepancy.

The anti-inflammatory and protecting gut integrity effects of LJE and LJE-Fs were comparable in human epithelial cells based on their capacity to attenuate LPS-induced NO production, as well as TEER, results in this study (Figure 3 and Figure 4). High levels of NO are involved in the pathophysiological status of various diseases; its uncontrolled production can impair target tissues during the inflammation process [50]. LPS-induced NO production was significantly inhibited by pretreatment with LJE and LJE-Fs in Caco-2 cells, suggesting their capacity to reduce inflammation. In addition, LPS-induced IL-6 levels were also significantly reduced by LJE treatment, and even further reduction was observed in the groups treated with LJE-Fs in support of the anti-inflammatory effects of LJE and LJE-Fs in Caco-2 cells. However, LPS-induced TNF-α production was only significantly inhibited by LJE and LJE-F1 but not by LJE-F2 and F3 in the present study, which may suggest LJE and LJE-Fs differentially regulate TNF-α production by LPS stimulus. Furthermore, we evaluated functional gut permeability by measuring TEER, resulting in a protective effect by all four LJ extracts on the permeability of the LPS-induced Caco-2 cell monolayer. Ko et al. (2014) reported that the administration of LJ water extract (300 mg/kg) improved colitis signs, such as colon length, histological score, and IL-1β, IL-6, and TNF-α production in an in vivo dextran sodium sulfate-induced colitis model [39]. In fact, patients with IBDs appeared to have significantly increased TNF-α and IL-6 levels [49]; therefore, blocking and/or reducing TNF-α and IL-6 signaling has been proposed to be an effective treatment for IBDs. Therefore, our finding of LJE and LJE-Fs’s capacity to reduce LPS-induced NO and IL-6 in Caco-2 cells could support their potential beneficial effects on gut health, including their potential as an alternative treatment for IBs, although further study is warranted to dissect the different responses to TNF-α reduction among LJ extracts in the present study.

Furthermore, bioactive compounds of LJ water extracts, such as fucoidans, could in part explain the anti-inflammatory and gut barrier protective effects of LJ extracts in Caco-2 cell monolayers observed in the present study. Fucoidan extracted from LJ is a combination of various polysaccharides, mainly made of fucose, galactose, and sulfate, with smaller amounts of mannoses, glucuronic acid, glucose, rhamnose, arabinose, and xylose [51]. In detail, Hwang et al. (2016) demonstrated that low-molecular-weight fucoidan, the sulfated polysaccharides extracted from *Sargassum hemiphyllum* (100 μg/mL), significantly decreased the inflammation of the intestinal barrier by inhibiting IL-1β and TNF-α and promoting IL-10 and IFNγ in Caco-2 cells [52]. Iraha et al. (2013) demonstrated that fucoidan isolated from *Cladosiphone okamuranus Tokida* protected gut integrity via regulation of claudin-1 in H_2_O_2_-stimulated Caco-2 cells [11]. Thus, both a crude water extract of LJ and LJ-derived fucoidans reduced inflammation and protected the intestinal barrier.

Occludin is an integral membrane protein that is specifically located at the TJs in the epithelia [49]. Although the function of occludin is unclear, a few studies using in vivo and in vitro models demonstrated the important roles of occludin in the TJ structure and permeability in the intestinal epithelia [49,53]. Unlike claudins, which mainly affect the flux of smaller-sized molecules and ions through a fixed pore, occludin plays an important role in the flux of large macromolecules, such as inulin and dextran, in the paracellular barrier [53]. LJE and LJE-Fs were able to block the inhibition of the protein expression of occludin by LPS stimulus in Caco-2 cells in the present study (Figure 5). Presumably, the anti-inflammatory effect of LJE and LJE-Fs is partially due to the enhancement of occludin expression, which is crucial to the selective flux of macromolecules, including bacterial antigens. Lastly, cumulative evidence supports the notion that a well-known energy sensor, AMPK, indeed has beneficial effects on gut health by increasing nutrient absorption, decreasing intestinal inflammation, and improving gut barrier function [38]. Among the four LJ extracts, pretreatment with LJE, LJE-F1, and LJE-F3 significantly attenuated the reduction in phosphorylation of AMPK in the LPS-stimulated Caco-2 cell monolayer. In summary, all four LJ extracts significantly prevented the reduction in the protein expression of occludin, whereas LJ, LJE-F1, and LJE-F3 attenuated the inhibition of the phosphorylation of AMPK compared with the LPS-treated group in Caco-2 cells, suggesting that the gut-integrity-protective effects of LJE and LJE-Fs are partially regulated by occludin and AMPK activation.

In conclusion, LJ water extract and three types of fermented LJ extracts by probiotic strains LGG, BB-12, or a combination of both, appeared to have potential gut-health-promoting effects by reducing intestinal inflammation and promoting the gut barrier function by partially regulating the tight junction-related proteins occludin and AMPK in human intestinal epithelial cells. A major limitation of our study is that the regulation of intestinal barrier function cannot be elucidated using an in vitro model alone due to the complexity of the intestinal barrier. In addition, further study is warranted to clarify the more precise mechanism(s) of LJEs by evaluating a diverse range of inflammatory cytokines, as well as more TJ-related proteins. Nevertheless, we believe that our findings support the potential benefit of LJE and LJE-Fs as an appropriate therapeutic agent for the treatment of inflammatory bowel diseases.

## Figures and Tables

**Table 1:** Changes of pH, total sugar contents, reducing sugar content, and total polyphenol contents in LJE-Fs by fermentation.

Measurements	Fermentation	LJE-F1	LJE-F2	LJE-F3
pH	Before	7.30 ± 0.12 ^1^	7.31 ± 0.14	7.34 ± 0.10
	After	4.06 ± 0.09 *	4.01 ± 0.11 *	3.95 ± 0.15 *
Total sugar (mg D-glucose/mL)	Before	4.40 ± 0.13	4.67 ± 0.02	4.93 ± 0.01
	After	3.34 ± 0.06 *	4.35 ± 0.07 *	4.04 ± 0.01 *
Reducing sugar (mg D-glucose/mL)	Before	1.44 ± 0.03	1.39 ± 0.01	1.47 ± 0.03
	After	1.39 ± 0.09 *	1.36 ± 0.03 *	1.36 ± 0.03 *
Total polyphenols (μg gallic acid/mL)	Before	1.14 ± 0.01	1.15 ± 0.07	1.17 ± 0.01
	After	0.90 ± 0.01	1.01 ± 0.01	1.11 ± 0.04

^1^ Values are mean ± SEM of three independent experiments. * Values with an asterisk are significantly different by *t*-test (before vs. after fermentation, *p* < 0.05). Abbreviations: LJE-F1, LJE fermented by LGG; LJE-F2, LJE fermented by BB-12; and LJE-*F3*, LJE fermented by a combination of LGG and BB-12.

**Table 2:** Effect of LJE and LJE-Fs on TNF-α and IL-6 production in the LPS-stimulated Caco-2 cells.

Sample	TNF-α Levels (pg/mL)	IL-6 Levels (pg/mL)
LPS	21.75 ± 0.27 ^b,1^	5.47 ± 0.7 ^c^
LPS+LJE	18.36 ± 1.20 ^a^	4.16 ± 0.26 ^b^
LPS+LJE-F1	18.16 ± 0.01 ^a^	0.92 ± 0.15 ^a^
LPS+LJE-F2	19.82 ± 0.19 ^b^	0.91 ± 0.03 ^a^
LPS+LJE-F3	19.11 ± 0.60 ^b^	0.77 ± 0.05 ^a^

^1^ Values are mean ± SEM of three independent experiments. Values that do not share the same superscript are significantly different by one-way ANOVA with Tukey’s comparisons test (*p* < 0.05). Caco-2 cells were pre-incubated for 6 h with 100 μg/mL LJE or LJE-Fs and then incubated for 24 h in the absence or presence of LPS (100 μg/mL).

## Abbreviations

AMPK	adenosine monophosphate (AMP)-activated protein kinase
BB-12	*Bifidobacterium animalis* ssp. *lactis* BB-12
IBD	inflammatory bowel diseases
LGG	*Lactobacillus rhamnosus* LGG
LJ	*Laminaria japonica*
LJE	*Laminaria japonica* extract
LJE-Fs	Fermented *Laminaria japonica* extract
LPS	lipopolysaccharides
NO	nitric oxide
TEER	transepithelial electrical resistance
TJ	tight junction
